# Novel 3D printed integral customized acetabular prosthesis for anatomical rotation center restoration in hip arthroplasty for developmental dysplasia of the hip crowe type III: A Case Report

**DOI:** 10.1097/MD.0000000000022578

**Published:** 2020-10-02

**Authors:** Heng Zhang, Yang Liu, Qirong Dong, Jianzhong Guan, Jiansheng Zhou

**Affiliations:** a Department of Orthopedics, the First Affiliated Hospital of Bengbu Medical College, Laboratory of Tissue and Transplant in Anhui Province, Bengbu Medical College, Bengbu City, Anhui Province; b Department of Orthopedics, the Second Affiliated Hospital of Soochow University, Suzhou City, Jiangsu Province, China.

**Keywords:** 3-dimensional, arthroplasty, customized prosthesis, hip dysplasia, rotation center

## Abstract

**Rationale:**

Exact restoration of the rotation center in total hip arthroplasty (THA) is technically challenging in patients with end-stage osteoarthritis due to developmental dysplasia of the hip (DDH), especially in the Crowe type II and III procedures. The technical difficulty is attributable to the complex acetabular changes. In this study, a novel 3-dimensional (3D) printed integral customized acetabular prosthesis for anatomical rotation restoration in THA for DDH Crowe type III was developed using patient-specific Computer-aided design and additive manufacturing (AM) methods.

**Patient concerns:**

A 69-year-old female patient had developed left hip joint pain and restricted movement for 40 years; the symptoms had increased in the past 5 months. Pain, limited motion of the left hip joint, and lower limb length discrepancy were noted during physical examination.

**Diagnosis:**

The patient was diagnosed with left hip end-stage osteoarthritis secondary to DDH (Crowe type III).

**Intervention:**

A 3D printed acetabulum model was manufactured and a simulated operation was performed to improve the accuracy of reconstruction of the rotation center and bone defect. A 3D printed titanium alloy integral customized acetabular prosthesis was designed according to the result of simulated operation. The integral customized prothesis was implanted subsequently via the posterolateral approach. Radiography of the pelvis and Harris score assessment were performed during the perioperative period as well as at the 6- and 12-month follow-up.

**Outcomes:**

The 3D printed integral customized acetabular prosthesis matched precisely with the reamed acetabulum. The rotation center was restored and the bone defect was exactly reconstructed. There were no signs of prosthetic loosening at the 12-month follow-up. The Harris score gradually improved during the follow-up period.

**Lessons:**

Satisfactory results of hip rotation restoration and bone defect reconstruction could be achieved by using 3D printed integral customized acetabular prosthesis, which provides a promising way to reconstruct the acetabulum in patients with DDH anatomically and rapidly for THA.

## 1 Introduction

The acetabular pathomorphology of developmental dysplasia of the hip (DDH) is complicated. It mainly presents as follows: the acetabular socket is shallow and flat, the hip rotation center migrates in the cephalic and lateral directions, a large number of osteophytes proliferate, and the anterior and posterior walls of the acetabulum are poorly developed. In cases of high dislocation, false acetabula appear above true acetabula, which can be difficult to distinguish.^[1]^ These acetabular changes pose challenges to orthopedic surgeons regarding correctly installing an acetabular prosthesis in total hip arthroplasty (THA), especially in rotation center restoration and bone defect reconstruction.^[2]^ Many difficulties in reconstructive surgeries have been solved with the development of 3D (3-dimensional) printing technology,[^3^,^4^] which can be applied in both preoperative planning and intraoperative patient-specific prothesis fabrication; it has been reported to yield good outcomes, at least at the short-term follow-up.^[5]^

In our previous study, we developed a method to restore the hip rotation center successfully in THA for DDH of Crowe type I-IV.^[6]^ We used this method to simulate an operation on a 3D printed acetabulum model on the basis of patient's 3D CT (computed tomography) data in this study. The cup size and bone defect volume were recorded and were used to design a patient-specific prothesis. The integral customized acetabular prosthesis with a wing part that matched the acetabular bone defect perfectly was made using electron beam melting technology. This enabled simultaneous anatomical reconstruction of the hip rotation center and bone defect. To the best of our knowledge, this is the first attempt on using 3D printing technology to fabricate a customized integral prosthesis to realize acetabular reconstruction anatomically in THA for DDH. Moreover, the integral acetabular prosthesis developed a larger contact interface between the host bone and the prothesis, providing greater bone in-growth potential. The success of the prosthesis design, fabrication, and surgical implantation in this case demonstrates the efficacy of this new method. We believe that 3D printed integral customized acetabular prothesis could be applied further for successful treatment of more challenging cases.

## 2 Case presentation

### 2.1 Ethical statement

This study was conducted in accordance with the principles outlined in the Declaration of Helsinki. This study was approved by the Ethics Committee on Human Research of the First Affiliated Hospital of Bengbu Medical College, and written informed consent was obtained from the patient and her family members for publication of this case report and accompanying images.

### 2.2 Clinical data

**Figure 1:**
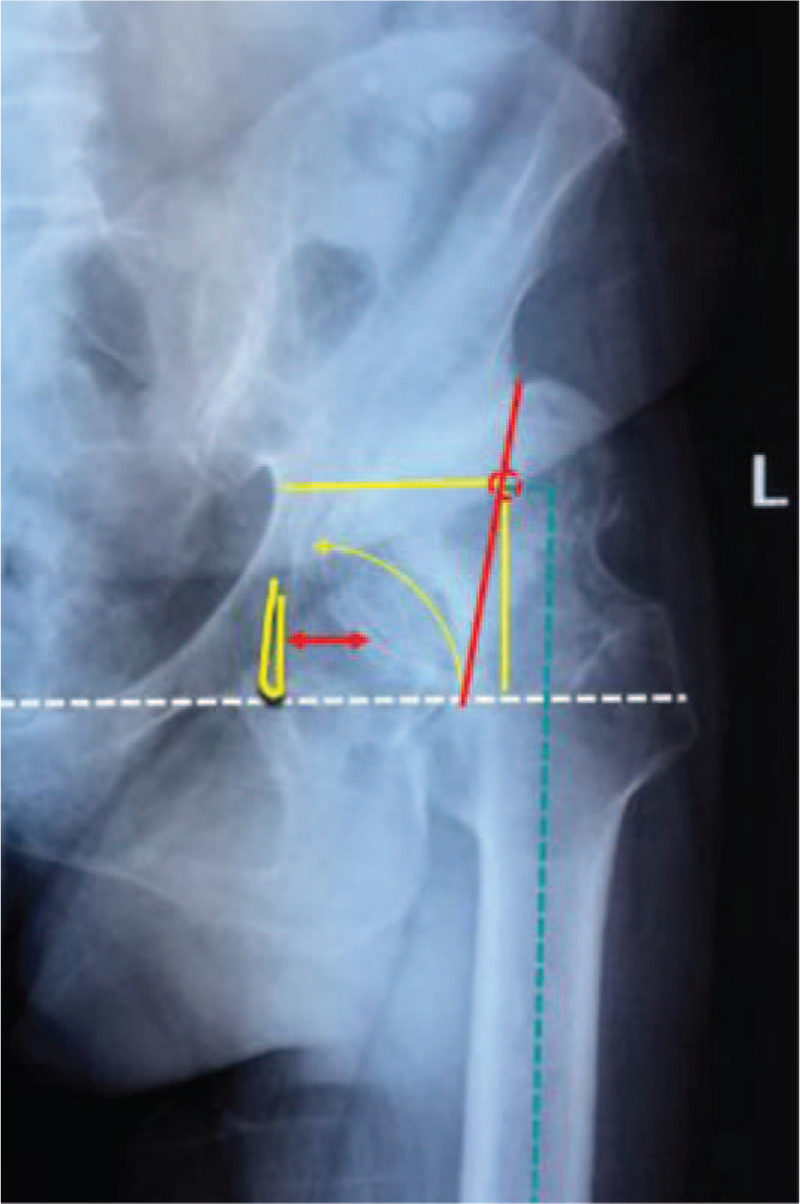
Preoperative X-ray.

**Figure 2:**
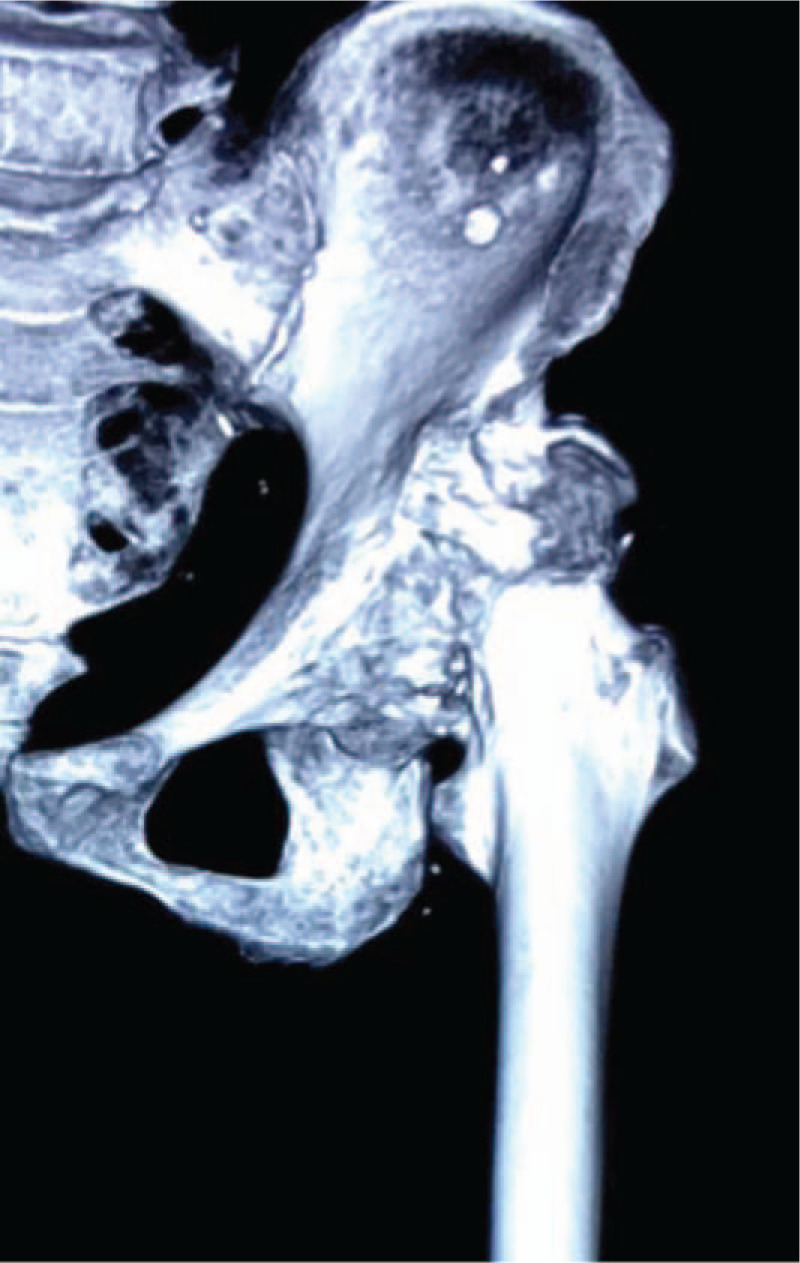
Preoperative 3-dimensional-computed tomography. 3D = 3-dimensional.

A 69-year-old female patient complained of left hip pain and limited mobility for 40 years, which had worsened significantly in the past 5 months. The left hip joint presented a flexion deformity. The range of motion of the left hip was as follows: forward flexion 25°, backward extension –10°, adduction 15°, and abduction 5°. The 4 sign was positive for the left hip joint. The lower limb length discrepancy was 3.6 cm. This patient was diagnosed with left hip end-stage osteoarthritis secondary to DDH (Crowe type III). The preoperative radiograph showed that the left acetabulum was superficial flat with high dislocation of the femoral head, the sharp angle was 79°, the Shenton line was interrupted, the joint space was narrow and disappearing, the surface of the Harris fossa was covered with a large number of osteophytes, the horizontal distance of the rotation center was 4.17 cm, the vertical distance was 4.97 cm, and the femoral offset was 0.98 cm (Fig. 1). The false socket and osteophytes could be clearly visualized on the computed tomography image (Fig. 2).

### 2.3 Preoperative simulated experiment

**Figure 3:**
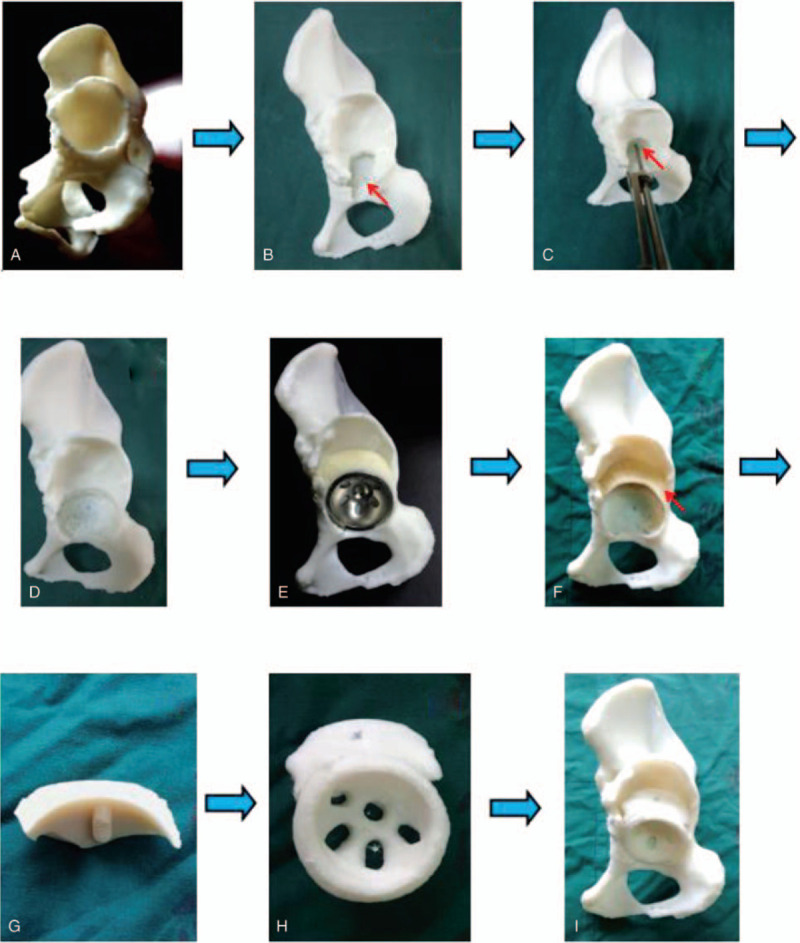
(A) Three-dimensional printing model. (B) restoring Harris fossa. (C) locating the acetabular center by acetabular center locator. (D) Reaming the acetabulum. (E) Installing the acetabular cup. (F) Filling the bone defect with bone wax. (G) The size of bone wax (bone defect). (H) Integral customized acetabular prothesis model. (I) Restoring the acetabulaum exactly. 3D = 3-dimensional, ACL = acetabular center locator.

The left hip joint model was printed using a 3D printing machine (Arigin 3DM400, Shanghai Arigin Medical Technology Co. LTD, China) using polylactic acid (PLA) material (Fig. 3A). The Harris fossa was covered with osteophytes; its morphology was restored by removing the osteophytes (Fig. 3B). The acetabular center was located using a acetabular center locator we developed (Fig. 3C). The acetabular socket was reamed at 15° anteversion and 40° inclination from small to large, aiming at the acetabular center. Reaming was started at a concentric circle of the acetabular center and to the depth of the Harris fossa's bottom (Fig. 3D). Considerable attention should be paid to the anterior and posterior walls of the acetabulum during the reaming process, especially the thickness of the anterior wall. The final cup size was determined according to whether the anterior and posterior walls of the acetabulum could maintain the stability of the cup (Fig. 3E). The bone defect above the cup was filled with bone wax (Fig. 3F). The size of the cup and bone defect were recorded (Fig. 3G). The model of the integral acetabular prosthesis was designed using UG software (Unigraphics NX by Siemens PLM Software, Germany) and printed using the 3D printing machine (Fig. 3H). The model of the integral customized acetabular prosthesis fit the grind acetabulum exactly (Fig. 3I).

### 2.4 The design and manufacture procedures for the 3D printed integral customized acetabular prosthesis

**Figure 4:**
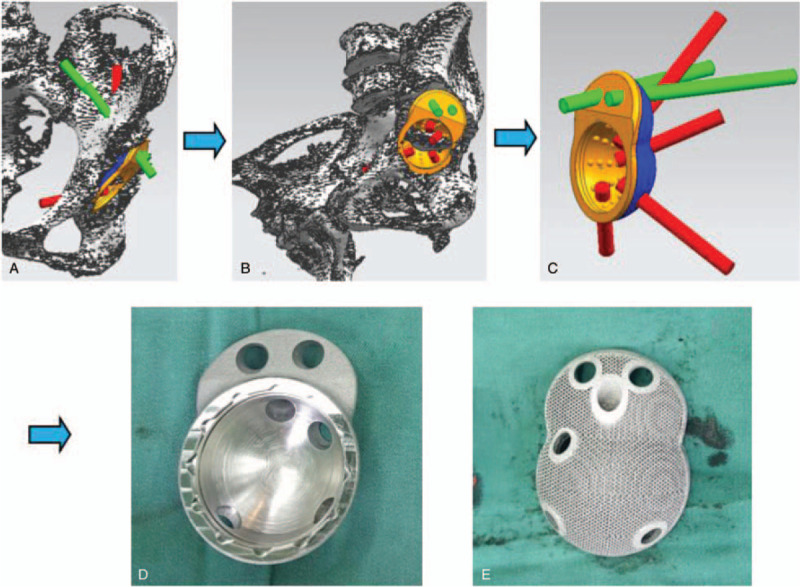
(A, B) Designing the integral customized acetabular prothesis. (C) The model of integral customized acetabular prothesis and screw directions. (D, E) 3-dimensional printing integral customized acetabular prosthesis. 3D = 3-dimensional.

The integral customized acetabular prosthesis was designed according to the result of preoperative simulative experiment performed using UG software. Four screws (red) pointed to the pubis, ischium, and posterosuperior part of the ilium (Fig. 4A, B). Two screws (green) pointed to the sacroiliac joint (Figs. 4A and 4B). The blue area was designed using a 3D printed microporous structure on the bone-prosthesis interface to promote bone in-growth and long-term prosthetic stability. Ti6Al4 V was chosen as the material for the prosthesis (yellow part) (Fig. 4C). The integral acetabular titanium alloy prosthesis was manufactured using the Arcam A1 3D print machine (Arcam Corporation, Beijing, China) by means of the electron beam melting 3D printing technique (Fig. 4D and E).

### 2.5 Surgical procedure

**Figure 5:**
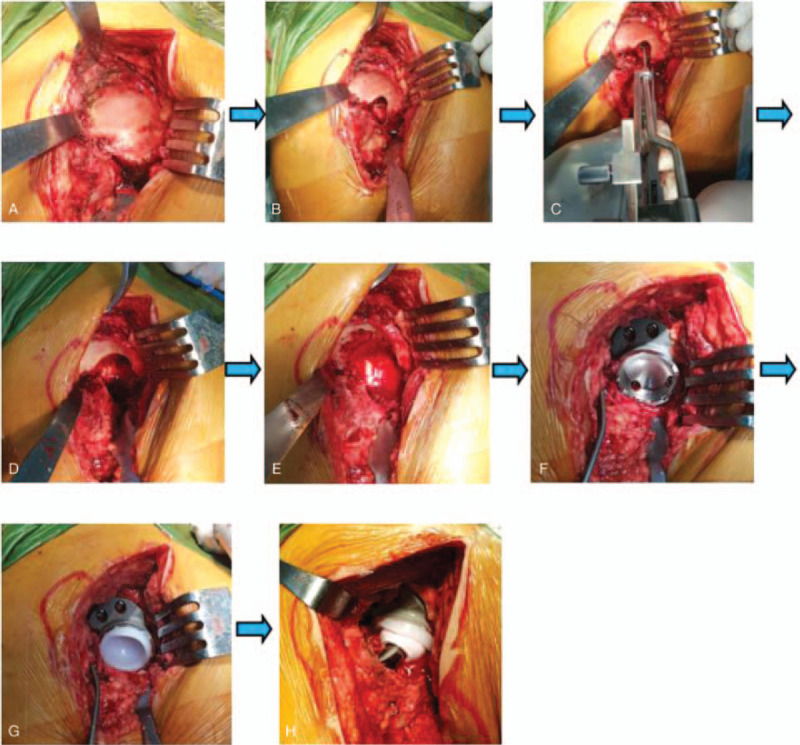
(A) Exposing the acetabulum. (B) Restoring Harris fossa. (C) Locating the acetabular center by acetabular center locator. (D) Reaming the acetabulum. (E) Preparing bone bed(bone defect area). (F) Installing integral customized acetabular cup. (G) Installing polythylene liner. (H) Installing hip joint prothesis. 3D = 3-dimensional, ACL = acetabular center locator.

The operation was performed via the posterolateral approach with the patient in the lateral decubitus position. The femoral head, femoral neck, and proximal femur were exposed and the sciatic nerve was carefully protected. The contracture soft tissue around the hip was released completely. Soft tissue and fat were cleared from the true acetabulum, which was identified by tracing and resecting the elongated and thickened joint capsule. The true acetabulum was exposed completely by placing 3 Hoffman retractors in the front, in the rear, and at the bottom of the acetabulum, respectively (Fig. 5A). The Harris fossa's morphology was restored by removing the osteophytes (Fig. 5B). The acetabular center was located using an acetabular center locator we developed (Fig. 5C). The acetabular socket was reamed at 15° anteversion and 40° inclination from small to large, aiming at the acetabular center (Fig. 5D). The reaming was performed at the concentric circle of the acetabular center and to the depth of the Harris fossa's bottom. Significant attention should be paid to the anterior and posterior walls of the acetabulum during the reaming process, especially the thickness of the anterior wall. The final size of the grind acetabulum was determined according to the criterion that the anterior and posterior walls of the acetabulum should have sufficient bone to maintain the stability of the cup prosthesis. The bone defect area was drilled to promote bone ingrowth (Fig. 5E). We inserted the integral customized acetabular prothesis via press-fit fixation, although additional screws were applied to reinforce the initial stability of the cup (Figs. 5F, 5G, and 5H).

### 2.6 Postoperative Management

Antibiotics were administered for infection prophylaxis within 24 hours. Anticoagulants were used to prevent deep vein thrombosis for 5 weeks. Ankle and quadriceps contraction exercises were started from the first day after operation. The patient could perform touch-down weight bearing under the assistance of crutches until 4–6 weeks after the operation, followed by partial weight bearing until 12 weeks, and full weight bearing thereafter.

**Figure 6:**
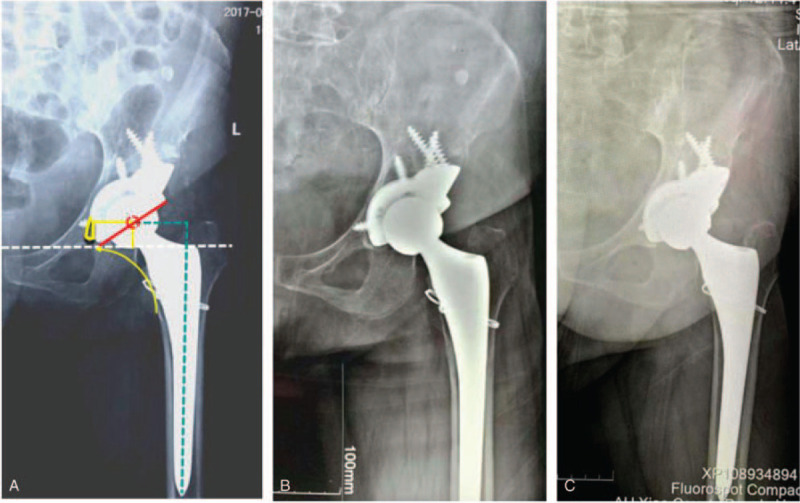
(A) Postoperative X-ray. (B) X-ray at 6-month follow-up. (C) X-ray at 12-month follow-up.

The patient was followed up clinically and radiologically at 0 week, 6 months, and yearly thereafter (Figs. 6A, 6B, and 6C). The total duration of follow-up has been 12 months up to now. Harris score was used to evaluate the recovery of hip joint function. Radiological assessment was performed using the pelvis plain radiograph. The vertical distance from the center of hip rotation to the inter-teardrop line and the horizontal distance from the center of hip rotation to the ipsilateral teardrop were measured, which were used to assess the efficacy of restoring the hip rotation center and acetabular cup migration.

## 3 Results

The Harris score improved from 31 points preoperatively to 92 points postoperatively. The horizontal distance of the rotation center was 3.52 cm, the vertical distance was 1.93 cm, and the femoral offset was 4.34 cm (Fig. 6A). The hip rotation center and bone defect were reconstructed anatomically. The press-fit between the customized prothesis and acetabulum was perfect. No acetabular cup migration and radiolucent line were found until the last follow-up (Fig. 6C).

## 4 Discussion

The changes of acetabular morphology in adult DDH are complicated. The pathological changes mainly include: a shallow and flat acetabular socket, upward and outward migration of the hip rotation center, as well as poor development of the anterior and posterior wall of the acetabulum. Thereafter, it is difficult to distinguish the false acetabulum and the true acetabulum based solely on the appearance. However, the acetabular morphological deformity in Crowe type III is more obvious than those in Crowe types I, II, and IV, resulting in greater difficulty of acetabular reconstruction and the installation of acetabular prosthesis in THA, especially for the restoration of the hip rotational center and the reconstruction of bone defect.^[7]^

At present, whether the hip rotation center in THA for Crowe type III DDH should be anatomically restored or highly restored is still controversial. Many scholars[^7^,^8^,^9^,^10^] recommended high restoration, because the host bone coverage rate of acetabular prosthesis could reach 70%, thus increasing the initial stability and promoting bone in-growth. Long-term stability of the acetabular prosthesis could be achieved with bone integration, but surgical procedures could be simplified. However, the biological stress distribution of the hip joint is changed and the wear rate of the prosthesis interface is increased with the hip rotation center moving upward, which contribute to lower limb length discrepancy, acetabular impingement, gluteus medius fatigue, gait changes, and hip dislocation. However, some scholars[^11^,^12^,^13^] advocate anatomical restoration of the hip rotation center, as anatomical restoration could recover the balance between the prosthesis and soft tissue, thus reducing the wear rate and prosthesis loosening. Unfortunately, it is difficult to locate the rotation center during the operation. Many methods, such as the template method, concentric circle method of the healthy side, Ranawat method, Pierchon method, and Pagnano method have been used to locate the rotation center of the hip joint. However, the above methods are all determined using a 2-dimensional image of the pelvis. Surgeons lack an intuitive and operable method to locate the hip rotation center during the operation. Zhang et al^[6]^ reported that the acetabular center was located on average of 28 mm (range, 25–31 mm, depending on acetabulum size) above the vertical bisection of the line between the anterior and posterior acetabular notches; moreover, the acetabular center was at the cephalic side of the Harris fossa, near the semilunar cartilage. The acetabular center could be located accurately in THA for DDH using this method. In this case, it was easy for us to locate the acetabular center and restore the hip rotation center with the help of the acetabular center locater, which was invented according to this method.

Currently, the management methods of DDH acetabular bone defect in THA mainly include: granule bone graft, structural bone graft, tantalum metal augment, and bone cement technology.[^14^,^15^,^16^] Impaction granule bone graft is usually performed using layer impaction technology, which has high technical requirements for the surgeon and requires special instruments.^[14]^ Structural bone graft can provide good initial stability for the prosthesis, but there are high risks of bone resorption and collapse as well as prosthesis loosening in the long term.^[17]^Tantalum metal augment can effectively repair bone defects, but it is difficult to match with the bone defect shape perfectly and reconstruct bone defects accurately.^[18]^ Bone cement method is an older technique, which is only applicable in elderly patients with severe osteoporosis. It can increase the initial stability; however, the long-term outcome is poor.^[19]^ The development of 3D printing technology provides a new way to reconstruct DDH acetaular bone defects. According to the preoperative 3D CT data of the patient's acetabulum, an individualized 3D printing customized prothesis can be developed to realize a perfect match with the bone defect shape to reconstruct the bone defect accurately to the maximum. With the assistance of computer software, the internal screw directions of the prothesis can be precisely designed, making the reconstruction of bone defects more reliable and safer.

Here, we present a solution for the hip rotation center restoration and acetabular bone defect reconstruction accurately in THA for DDH Crowe type III using a 3D printing method. Importantly, the patient obtained a good outcome during our follow-up. Notably, this method enabled precise treatment and simplified the operation. Although the long-term result of this method is still unknown, we believe that 3D printed integral customized acetabular prothesis will have an important role in DDH THA, especially for Crowe type II and III cases.

## Author contributions

**Conceptualization:** Heng Zhang, Jianzhong Guan, Jiansheng Zhou.

**Data curation:** Heng Zhang, Yang Liu.

**Formal analysis:** Heng Zhang.

**Funding acquisition:** Heng Zhang, Jianzhong Guan, Jiansheng Zhou.

**Investigation:** Jianzhong Guan, Jiansheng Zhou.

**Methodology:** Heng Zhang, Jianzhong Guan, Jiansheng Zhou.

**Project administration:** Heng Zhang.

**Resources:** Heng Zhang, Jianzhong Guan.

**Software:** Heng Zhang, Jianzhong Guan.

**Supervision:** Jianzhong Guan, Jiansheng Zhou.

**Visualization:** Heng Zhang.

**Writing – original draft:** Heng Zhang.

**Writing – review & editing:** Heng Zhang, Qirong Dong.

**Writing:** Heng Zhang, Qirong Dong.
